# How fly neurons compute the direction of visual motion

**DOI:** 10.1007/s00359-019-01375-9

**Published:** 2019-11-05

**Authors:** Alexander Borst, Jürgen Haag, Alex S. Mauss

**Affiliations:** Max-Planck-Institute of Neurobiology, Martinsried, Germany

**Keywords:** Visual motion, Direction selectivity, *Drosophila*, Optic lobe, Preferred direction enhancement, Null direction suppression

## Abstract

Detecting the direction of image motion is a fundamental component of visual computation, essential for survival of the animal. However, at the level of individual photoreceptors, the direction in which the image is shifting is not explicitly represented. Rather, directional motion information needs to be extracted from the photoreceptor array by comparing the signals of neighboring units over time. The exact nature of this process as implemented in the visual system of the fruit fly *Drosophila melanogaster* has been studied in great detail, and much progress has recently been made in determining the neural circuits giving rise to directional motion information. The results reveal the following: (1) motion information is computed in parallel ON and OFF pathways. (2) Within each pathway, T4 (ON) and T5 (OFF) cells are the first neurons to represent the direction of motion. Four subtypes of T4 and T5 cells exist, each sensitive to one of the four cardinal directions. (3) The core process of direction selectivity as implemented on the dendrites of T4 and T5 cells comprises both an enhancement of signals for motion along their preferred direction as well as a suppression of signals for motion along the opposite direction. This combined strategy ensures a high degree of direction selectivity right at the first stage where the direction of motion is computed. (4) At the subsequent processing stage, tangential cells spatially integrate direct excitation from ON and OFF-selective T4 and T5 cells and indirect inhibition from bi-stratified LPi cells activated by neighboring T4/T5 terminals, thus generating flow-field-selective responses.

## Introduction

**Fig. 1:**
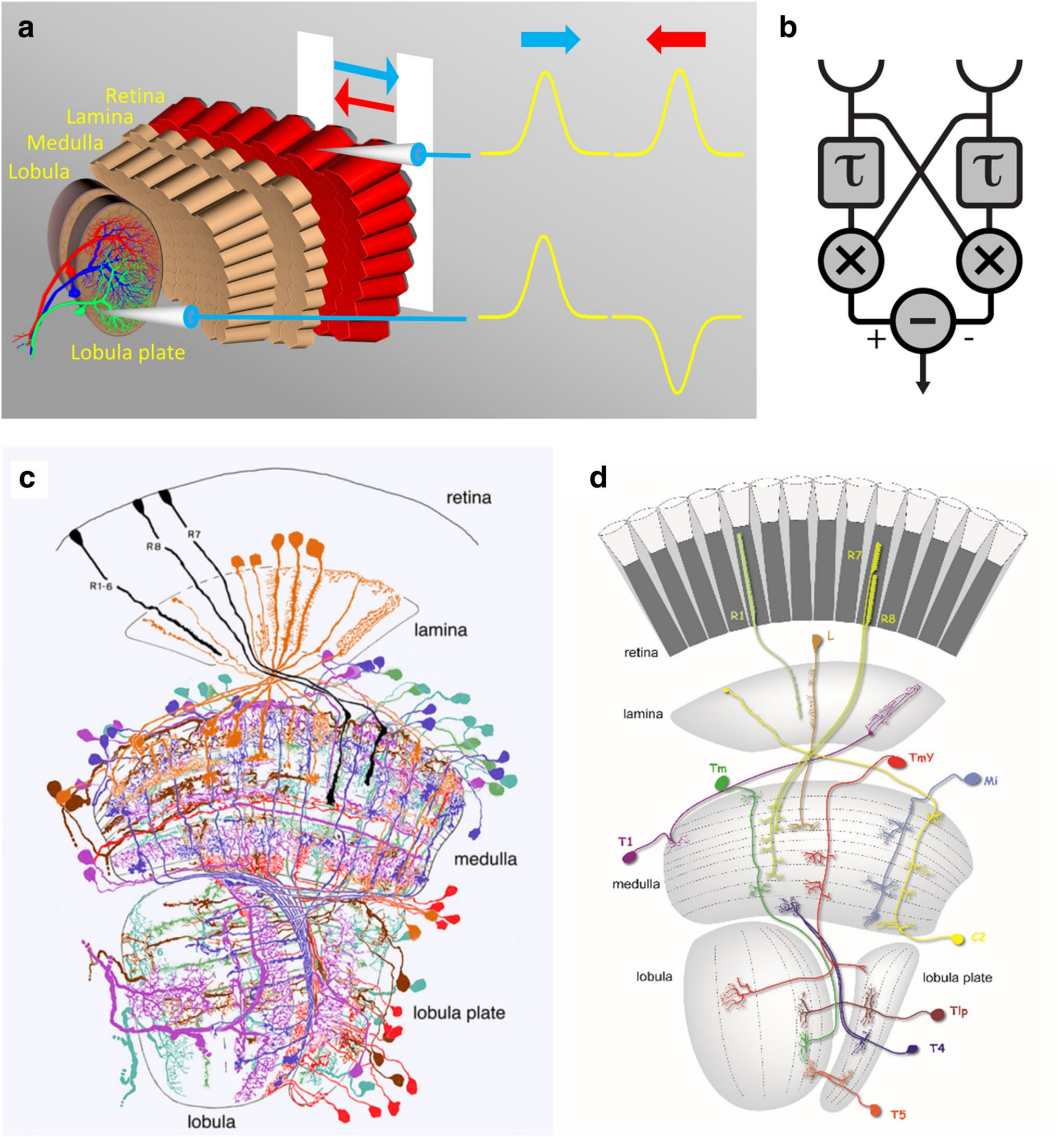
**a** Schematic illustration of the central phenomena of direction selectivity: moving a bar in front of the fly’s eye leads to a depolarization of photoreceptors each time, no matter whether the bar moves to the right or to the left. These signals are non-directional. Just a few synapses downstream, at the level of the lobula plate tangential cells, signals are direction-selective: These cells depolarize during motion along one, i.e., their ‘preferred’, and hyperpolarize during motion along the opposite, i.e., their ‘null’ direction. **b** Hassenstein–Reichardt model for elementary motion detection (*τ* low-pass filter; × multiplication). **c** Collection of all the different columnar cell types found in the *Drosophila* optic lobe (after Fischbach and Dittrich 1989). **d** Schematics of individual cell type classes (from Borst 2009)

The direction of motion is an essential visual cue: when crossing a street, the question whether a car is moving away or towards us will be decisive for our future. The same applies to the animal kingdom: be it a predator or a prey, seeing every slightest change of the direction in which an object is moving can be of utmost importance for survival. In addition, every sighted animal uses motion cues when navigating through the world. The movement of an observer causes the images of the world to move across its retinae in characteristic patterns. The distribution of local motion vectors is called ‘optic flow’. Since it depends on the direction of the observer’s movement as well as on the structure of the environment (Gibson 1950; Koenderink and van Doorn 1987), it can provide useful feedback signals. Yet, the direction in which a patch of image is moving is not explicitly represented by the activity of a single photoreceptor (Fig. 1a). Rather, direction-selective signals must be computed by downstream neural networks. How this computation is done has been the focus of intense research over many decades, making this process a prime example for neural computation.

In general, any circuit that extracts directional information from the photoreceptor signals needs to meet three essential requirements: (1) input from at least two spatially offset inputs, (2) asymmetric temporal filtering, and (3) non-linear interaction between the filtered outputs (Poggio and Reichardt 1973; Buchner 1984; Reichardt 1987; Borst and Egelhaaf 1989). Analyzing the turning tendency of the beetle *Chlorophanus viridis* walking on a spherical Y-maze, Hassenstein and Reichardt (1956) proposed a specific model of elementary motion detection that could account for their observations in a quantitative way. The idea was that the beetle’s nervous system contains many hundreds of such elementary units, which, collectively, cover the whole visual field, each extracting locally the direction of image motion. The model for such an elementary motion detector consists of two subunits, which are mirror-symmetrical to each other (Fig. 1b; Reichardt 1961, 1987; Borst and Egelhaaf 1989). Each subunit reads the luminance values measured in two adjacent facets (requirement 1). One of the inputs is shifted in time with respect to the other by a temporal low-pass filter (*τ*, requirement 2). Next, the signals become multiplied (*x*, requirement 3). This is the first stage at which a directionally selective signal arises. As a final processing step, signals of two subunits with opposite direction preference become subtracted, producing a fully opponent response: positive for motion in one direction and negative for motion in the opposite direction. The value of this model for research in the field of motion vision can hardly be overestimated. The reason for this is that this model makes predictions that are both quantitative and counterintuitive. This model is referred to in the literature as Hassenstein–Reichardt model (HRM), correlation detector, elementary motion detector (EMD), or Reichardt detector. Numerous studies at the behavioral level as well as at the level of large tangential cells have produced data that are consistent with the model (Goetz 1964; Buchner 1976; Borst and Egelhaaf 1989; Egelhaaf et al. 1989; Schuling et al. 1989; Borst et al. 2003; Haag et al. 2004; Clark et al. 2011).

A variant of the correlation-type movement detector has been proposed by Barlow and Levick to explain their experimental findings on directionally selective ganglion cells in the rabbit retina (Barlow and Levick 1965). With respect to its layout, the Barlow–Levick model is almost identical to one subunit of the basic Hassenstein–Reichardt model. It consists of two input lines carrying the brightness signals which are compared after one of the signals has been delayed. In contrast to the Hassenstein–Reichardt model, this comparison is accomplished by a special logical gate, an ‘and-not’ or ‘veto’ gate. This means that the detector’s activity is suppressed when both input signals arrive simultaneously at the ‘and-not’ gate. Note that the ‘and-not’ operation is equivalent to a division. The corresponding direction of motion is, therefore, the detector’s null direction.

## The fly visual system

The above-described models are purely algorithmic in nature. How are the computations implemented neuronally in the fly visual system? In the 1970s, large motion-sensitive neurons have been identified in the third visual neuropil of the optic lobe, the lobula plate (Fig. 1a) (Dvorak et al. 1975). Such neurons, termed lobula plate tangential cells (LPTCs), respond with depolarization to visual motion in their preferred direction and hyperpolarization to motion in the opposite direction (Hausen 1984), just like the subtraction stage of the fully opponent Reichardt detector. Some LPTCs, like for instance HS cells shown in Fig. 1a, are sensitive to horizontal motion. VS cells in contrast are selective for vertical motion. In addition to motion opponency, other response properties such as temporal frequency tuning or contrast dependence are also in precise agreement with visual signal processing according to a Reichardt model (Borst and Egelhaaf 1989). However, LPTCs integrate over large parts of the visual field, i.e., they do not compute the direction of motion locally. Therefore, naturally, the question arises which of their upstream neurons constitute the local motion detector(s)?

Regarding the neurons which transmit the information from the photoreceptors to the lobula plate tangential cells, an almost complete catalog has been worked out: starting with the work of Cajal and Sanchez (1915), the columnar cell types of the lamina, medulla, lobula, and lobula plate have all been identified and described on the basis of Golgi impregnations in the house fly (Strausfeld 1976) as well as in *Drosophila* (Fischbach and Dittrich 1989, Fig. 1c). Each lamina column (or cartridge, as it is usually referred to) contains a set of 12 cell types, connected to the photoreceptors either directly or indirectly (Meinertzhagen and O’Neil 1991; Tuthill et al. 2013). These lamina neurons connect the photoreceptors to specific layers of the medulla (Takemura et al. 2008, 2017). In the medulla, a single column houses more than 60 different cell types. Based on their anatomy, they can be clustered into different groups (Fig. 1d). Medulla intrinsic (‘Mi’) neurons connect different layers of the medulla to each other, trans-medulla (‘Tm’) neurons connect specific layers of the medulla to various layers in the lobula, and trans-medulla Y (‘TmY’) neurons connect specific layers of the medulla to various layers in the lobula and lobula plate. Importantly, the so-called bushy T cells connect medulla layer 10 (T4 cells) and the posterior layer of the lobula (T5 cells) to the four layers of the lobula plate. However, the small size of all these neurons made electrophysiological recordings difficult. Therefore, while a rather complete map of all columnar neurons was at hands for long, the visual response properties of most of them were completely unknown for a long time, representing a collection of ‘silent neurons’, the function of which could only be guessed.

## Visual motion pathways

**Fig. 2:**
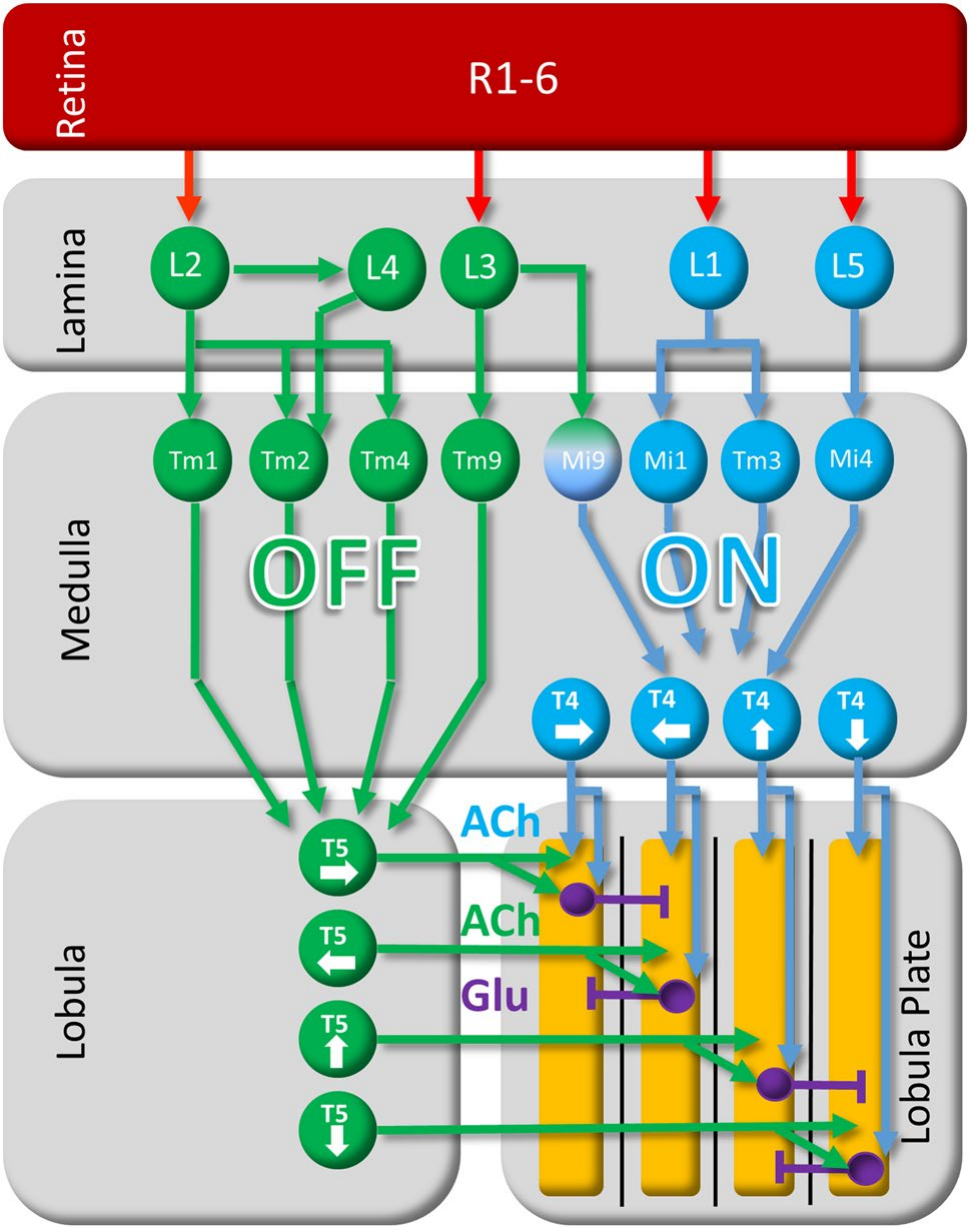
General layout of the neural circuit for motion vision in *Drosophila*. Note that the actual circuitry is more complex, in particular with respect to the medulla neurons involved as well as their synaptic interactions. *ACh* acetylcholine, *Glu* glutamate

It is for this reason that the problem of the neural implementation of the Hassenstein–Reichardt detector has been in the field for over half a century, becoming the ‘holy grail of fly motion vision’, with only little progress for many decades. Only the advent of sophisticated neurogenetic methods in *Drosophila* allowed for elucidating the circuits underlying elementary motion detection. These techniques are all based on a two-component expression system where a so-called ‘driver line’ defining the neurons where a certain effector gene is expressed is crossed with another line, the so-called ‘responder line’, defining what gene is expressed (Brand and Perrimon 1993). Today, thousands of different driver lines are available many of which reveal a high degree of specificity for expression in individual cell types of the optic lobe (Pfeiffer et al. 2008; Jenett et al. 2012; Tuthill et al. 2013). Furthermore, many responder lines have been developed to block the synaptic output of neurons, to activate neurons optogenetically as well as to record from neurons via genetically encoded calcium indicators (for review, see Borst 2009; Venken et al. 2011). Applying these techniques to the problem of local motion detection revealed the following picture (Fig. 2): (a) luminance information from fly photoreceptors R1–6 is split into two parallel motion circuits, specialized to detect the motion of luminance increments (ON channel) and decrements (OFF channel) separately (Joesch et al. 2010; Eichner et al. 2011; Joesch et al. 2013; Strother et al. 2014; Behnia et al. 2014). (b) Within each channel, T4 and T5 cells are the elementary motion-sensing neurons (ON- > T4, OFF- > T5). There exist four subtypes per column, each tuned to one of the four cardinal directions (Maisak et al. 2013). (c) According to their preferred direction, axon terminals of T4 and T5 cells make excitatory connections onto the dendrites of tangential cells within one layer of the lobula plate. There, they also excite lobula plate intrinsic neurons, which inhibit tangential cells in the adjacent layer (Mauss et al. 2014, 2015).

### Two parallel ON and OFF channels

As a first characteristic of the visual processing and in striking parallel to the mammalian retina (Schiller et al. 1986; Masland 2012), for review see: Borst and Helmstaedter 2015), the luminance signal becomes split into two pathways, and the direction of motion is computed in parallel within each of them. The ON pathway transmits information about brightness increments, while the OFF pathway deals with brightness decrements. Based on the anatomy of individual medulla neurons, their co-stratification in specific layers, and 2-deoxy-glucose activity labeling (Bausenwein and Fischbach 1992; Buchner et al. 1984), parallel motion pathways had been suggested for long, but their functional segregation was a matter for speculation. Only genetic targeting and blocking of specific lamina neurons allowed for resolving this issue. Blocking the output from lamina neurons L1, Joesch and colleagues found a specific response reduction of lobula plate tangential cells to brightness increments, while blocking the output of lamina neurons L2 reduced their response to brightness decrements (Joesch et al. 2010). These results were later confirmed in a study on behavioral responses of walking fruit flies (Clark et al. 2011). More recent evidence from blocking experiments as well as anatomy demonstrates that lamina neurons L3 (Silies et al. 2013; Shinomiya et al. 2014) and L4 (Takemura et al. 2011; Meier et al. 2014) provide additional contribution to the OFF pathway. Together, these experiments suggest that the photoreceptor input from R1-6 is split into parallel channels depending on the contrast polarity of the incoming signal: While the L1 pathway specifically transmits information about brightness increments to downstream motion detectors, the L2-4 pathway conveys information about brightness decrements (Fig. 2).

The question then arises how ON and OFF signals are combined further downstream to generate direction-selective responses. Theoretically, there could be four channels comprising all possible combinations (ON–ON, ON–OFF, OFF–OFF, and OFF–ON). However, apparent motion experiments in blow flies indicated that only two such channels exist, one for the interaction of ON signals and one for the interaction of OFF signals between neighboring image points (Riehle and Franceschini 1984). In these experiments, pairs of single photoreceptors were stimulated by light-ON and light-OFF flashes while recording from the motion-sensitive lobula plate neuron H1. Recordings from lobula plate tangential cells in *Drosophila* arrived at the same conclusion: only sequences of light pulses of the same contrast polarity (ON–ON and OFF–OFF) elicited significant responses, while light pulses of differing contrast polarity (ON–OFF and OFF–ON) failed to evoke any responses in lobula plate tangential cells (Eichner et al. 2011). Interestingly, using brightness steps instead of brightness pulses led to different results: while again, ON–ON and OFF–OFF sequences along the preferred direction of the cell led to positive responses, ON–OFF and OFF–ON sequences elicited negative responses. This result is related to the so-called ‘reverse phi’ phenomenon, which describes the illusion of opposite to actual motion when the contrast of a moving pattern is inverted every other frame (Anstis and Rogers 1975). This phenomenon was previously taken as evidence for an interaction between ON and OFF stimuli (Egelhaaf and Borst 1992; Clark et al. 2011; Tuthill et al. 2011), as in the original Hassenstein–Reichardt detector. However, careful simulations of such circuits as well as experiments with flies with either lamina neurons L1 or L2 blocked revealed that such an inversion of the response is not necessarily indicative of an interaction between ON and OFF channels. Rather, it can be explained by residual sustained response components of the neurons providing input to downstream motion-sensing neurons (Eichner et al. 2011; Joesch et al. 2013; Leonhardt et al. 2017). On the other hand, Salazar-Gatzimas et al. (2018) have recorded T4 and T5 calcium signals to reverse phi stimuli and found positive responses to specific ON–OFF (for T5) and OFF–ON (for T4) pairings in the null direction. The implication is that ON and OFF signals do interact at the level of local motion detectors, though not in a multiplicative way as in the original Reichardt detector which would produce negative responses.

### T4 and T5 cells

**Fig. 3:**
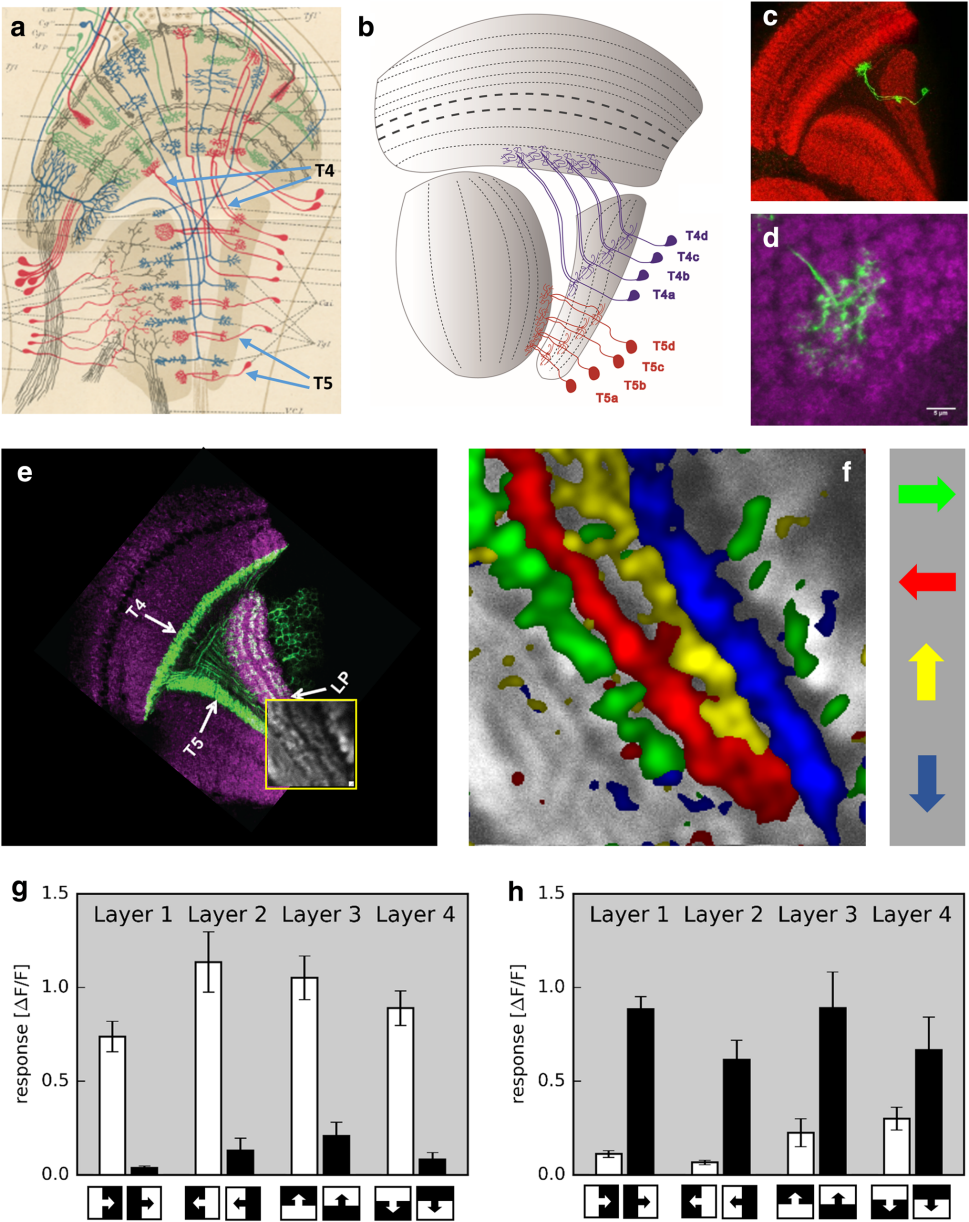
T4 and T5 cells are the elementary, motion-sensing neurons in the fly visual system. **a** First drawing of T4 and T5 cells (Cajal and Sanchez 1915; ‘T4’ and ‘T5’ labels and arrows added). **b** Schematic of the four types of T4 and T5 cells (after Fischbach and Dittrich 1989). **c** Individual T4 cell (Schilling and Pujol-Marti, unpublished). **d** Dendrite of an individual T4 cell extending across multiple medulla columns (Haag et al. 2016). **e** Confocal image of the optic lobe of a driver line, expressing in T4 and T5 cells (Maisak et al. 2013). **f** Calcium imaging reveals four subtypes tuned to four cardinal directions, each projecting to one of the four layers of the lobula plate (Maisak et al. 2013). **g, h** T4 cells (**g**) respond preferentially to moving ON edges; T5 cells (**h**) preferentially to moving OFF edges (data from Maisak et al. 2013)

Having identified lamina neurons L1 and L2 as important input elements to two parallel motion detector circuits allowed anatomy to guide the next steps. Indeed, two parallel processing streams had been postulated previously, based on careful investigation of co-stratification of Golgi-stained columnar cells (Bausenwein et al. 1992), as well as cell-unspecific activity labeling using the deoxy-glucose method (Bausenwein and Fischbach 1992). These studies indicated that an L1 pathway should indirectly lead to columnar T4 cells, and an L2 pathway to columnar T5 cells, with both T4 and T5 cells projecting into the lobula plate. T4 and T5 cells were first described by Cajal and Sanchez (1915) (Fig. 3a). There exist four different subtypes of T4 and T5 cells (termed a, b, c, and d) per column each of which terminates in one of the four different layers of the lobula plate (Fischbach and Dittrich 1989; Fig. 3b–d). Since 2-deoxy-glucose labeling indicated activity in one of the four layers according to the direction of the stimulus (Buchner et al. 1984), T4 and T5 cells were prime candidates for local elementary motion detectors and for representing the output signals of the ON and the OFF motion pathway, respectively. Their small size, however, prohibited electrophysiological recordings and let their visual response properties be unknown for long. This problem was solved using driver lines specific for T4 and T5 cells and combining them with a high-sensitivity genetically encoded calcium indicator GCaMP5 (Akerboom et al. 2012). Using 2-Photon calcium imaging and stimulating the flies with grating motion in four cardinal directions (front-to-back, back-to-front, upwards, downwards), Maisak and colleagues recorded direction-selective activity from T4/T5 cells, encoding a different direction in each lobula plate layer (Maisak et al. 2013; Figure 3e, f). To assess the particular contribution of T4 and T5 cells to the signals observed in the above experiments, driver lines specific for T4 or T5 cells were used, respectively. Applying the same stimulus protocol and data evaluation as before, identical results were obtained for both the T4- as well as the T5-specific driver line. Further experiments revealed similar response properties for T4 and T5 cells with respect to their orientation and velocity tuning (Maisak et al. 2013). If, however, instead of gratings, moving edges with either positive or negative contrast polarity were used as visual stimuli, T4 cells were found to strongly and selectively respond to moving ON edges, with little or no responses to moving OFF edges (Fig. 3g), while T5 cells selectively responded to moving OFF edges and mostly failed to respond to moving ON edges (Fig. 3h).

To investigate whether the specific response properties of T4 and T5 cells are visible in postsynaptic lobula plate cells and visually driven behavior, T4 and T5 cells were genetically blocked and flies subsequently tested. Blocking both T4 and T5 cells led to a complete loss of the motion response in lobula plate tangential cells (Schnell et al. 2012), of the optomotor turning response of tethered walking flies (Bahl et al. 2013), of walking speed modulation by translational flow (Creamer et al. 2018) as well as of the two types of looming-sensitive behaviors, i.e., the landing and the avoidance response (Schilling and Borst 2015). Blocking T4 cells specifically led to selective loss of the responses to moving ON edges, in the electrical signal of tangential cells as well as in optomotor behavior. Conversely, blocking T5 cells led to a loss of the responses to moving OFF edges in both assays (Maisak et al. 2013). In summary, the selective defects of T4 block flies for ON and of T5 block flies for OFF edges corroborate the above findings about the selective T4 and T5 cell edge responses. Furthermore, the behavioral defects of flies with both T4 and T5 cells blocked suggest that T4 and T5 cells are indeed the elementary motion-sensing neurons of the fly visual system, providing the major, if not exclusive, directional inputs to downstream circuits and motion-driven behaviors.

### Motion opponency by intrinsic lobula plate cells

**Fig. 4:**
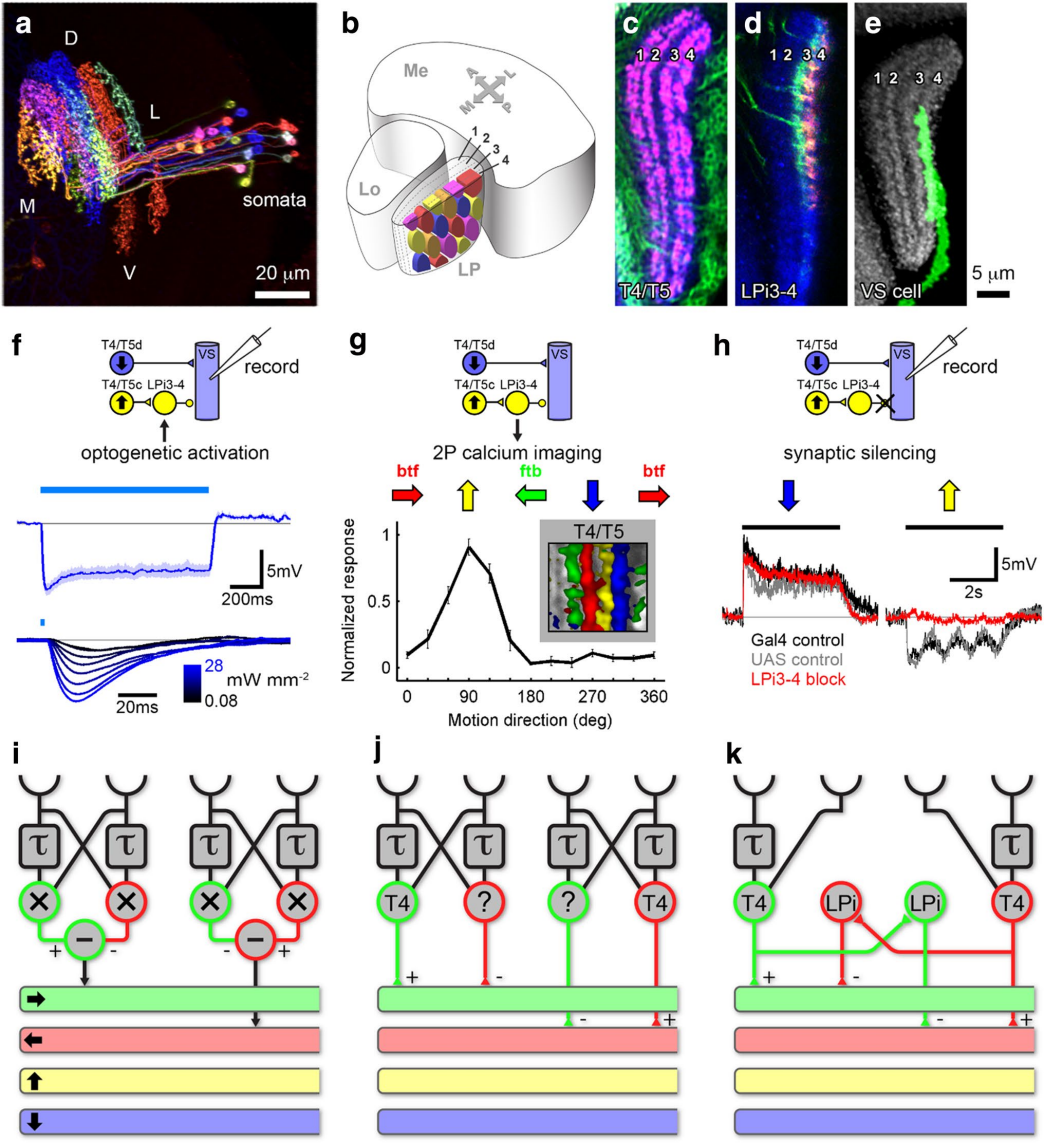
Intrinsic lobula plate neurons (LPi) implement the subtraction of opponent T4/T5 cells within the lobula plate. **a** Multicolor flip-out showing several individual LPi neurons tiling the lobula plate in visual space. **b** Schematic representation of the dendritic fields of adjacent LPi neurons. **c** Horizontal cross section of the lobula plate, with T4/T5 cells expressing GFP (green) and presynaptic synaptotagmin-HA (sytHA, red). The axon terminals form four layers. **d** LPi neurons expressing GFP (green) and presynaptic synaptotagmin-HA (sytHA, red). Neurons ramify in layers 3 and 4, but have presynaptic specializations restricted to layer 4 only. **e** A VS cell with downward direction selectivity extends its dendrite to layer 4. **f** The synaptic connection between LPi and VS cells probed functionally by optogenetic stimulation of LPi cells and patch-clamp recordings from VS cells. 1 s light stimulation of LPi neurons evokes a sustained hyperpolarizing potential (upper recording trace). 2 ms light stimulation elicits short latency hyperpolarizing responses that increase in amplitude with increasing light intensity (lower recording traces and light intensity in mW/mm^2^ color-coded from black-to-blue). **g** Visual activity in LPi3-4 neurons measured by 2P-calcium imaging. Normalized response is shown as a function of grating motion direction in degrees (deg). LPi3–4 neurons respond preferentially to upward motion, as do T4 and T5 cells terminating in layer 3. Note that red (btf: back-to-front) and green (ftb: front-to-back) arrows indicating motion direction are flipped compared to Fig. 3f, because experiments were done on different body sides. Left, this panel; right, Fig. 3f. **h** Visually evoked potential changes recorded via patch clamp from VS cells in three experimental fly strains: two controls (black and gray) with fully intact visual circuitry as well as flies with LPi3–4 neurons genetically silenced (red). VS cells in control flies exhibit motion-opponent responses, with depolarization in response to downward and hyperpolarization in response to upward motion. Blocking LPi cells leaves the depolarizing preferred direction response of tangential cells unaffected, but abolishes their hyperpolarizing null direction response. Recording traces over time represent the mean across several cells. *mV* millivolt, *s* second. **i–k** Relation of the algorithmic Hassenstein–Reichardt detector to the actual circuitry as found in the fly optic lobe. All panels except **c** and **i–k** adapted from Mauss et al. (2015). **c** adapted from Mauss et al. (2014). Inset in panel **g** adapted from Maisak et al. (2013)

Tangential cells depolarize in response to visual motion along their preferred direction and hyperpolarize during motion along the opposite direction. While the excitation during preferred direction can be readily explained by their excitatory, cholinergic input from those T4/T5 cells terminating within the lobula plate layer where the tangential cell extends its dendrite, the hyperpolarizing null direction response remained a mystery for quite some time. Does there exist another population of inhibitory elementary motion detectors? Two lines of evidence provided evidence that this is not the case. First of all, blocking T4/T5 cells not only abolishes the tangential cells’ preferred direction excitation, but at the same time also their null direction inhibition (Schnell et al. 2012). Second, optogenetic activation of T4/T5 cells leads to a biphasic postsynaptic potential in tangential cells, consisting of a fast excitation followed by a delayed inhibition (Mauss et al. 2014). Both of these findings suggest that T4/T5 cells not only excite the tangential cells, but also a feed-forward inhibitory element, subsequently inhibiting the tangential cells in the adjacent layer. Since the lobula plate is organized such that adjacent layers represent opposite directions of motion (Fig. 3f) rather than directions rotated by 90°, the existence of an inhibitory cell type was postulated that covers two adjacent layers with its processes (Mauss et al. 2014). Indeed, such bi-stratified neurons were found, termed Lobula *P*late intrinsic (LPi) cells (Fig. 4a, b; Mauss et al. 2015). Within the four layers formed by the axon terminals of T4/T5 cells (Fig. 4c), LPi cells ramify within exactly two adjacent layers, showing presynaptic specializations in only of them (Fig. 4d). When LPi cells are stimulated optogenetically, lobula plate tangential cells in layer 4 (Fig. 4e) reveal a fast, mono-synaptic, graded inhibitory postsynaptic potential (Fig. 4f). As shown by 2-Photon calcium imaging, LPi cells are directionally selective and have the same preferred direction as T4c/T5c cells, which terminate in their presumed postsynaptic layer (Fig. 4g). When blocked, tangential cells lose their hyperpolarizing response to null direction motion, while their preferred direction depolarization is unaffected (Fig. 4h). As is demonstrated by the above and other experiments, LPi cells receive T4/T5 input in one layer and convey an inhibitory glutamatergic signal to tangential cells expressing glutamate-gated Cl^−^ channel α in the neighboring motion-opponent layer (Mauss et al. 2015; Richter et al. 2018). Tangential cells thus integrate two sources of local direction-selective information: direct excitation from ON and OFF-selective T4 and T5 cells in joint lobula plate layers and indirect inhibition from bi-stratified LPi cells activated by neighboring T4/T5 terminals.

Looking back onto the original Hassenstein–Reichardt model, its neural implementation seems more parsimonious and differs in the following way: First, instead of performing the subtraction locally, before spatial integration (Fig. 4i), the subtraction occurs on the dendrites, simultaneously with spatial integration of directional motion information (Fig. 4j; Egelhaaf et al. 1989; Borst and Egelhaaf 1990). Second, instead of repeating the computation of a certain direction and edge polarity for its use as an inhibitory signal, this computation is done only once and subsequently converted into an inhibitory signal in the adjacent lobula plate layer via LPi neurons (Fig. 4k; Mauss et al. 2015).

What is the functional relevance of this subtractive processing stage? Due to their large receptive fields, lobula plate tangential cells are commonly thought to detect optic flow arising by self-motion (Krapp and Hengstenberg 1996; Krapp et al. 2001). Depending on the maneuver, flow fields may be unidirectional, as, e.g., experienced during body rotation about the left–right body axis, or they may be dominated by expansion, as e.g., occurring during forward translation. Recordings from VS cells show that they are predominantly depolarized by unidirectional (downward) and not expanding flow fields. Genetically silencing the LPi cells strongly impaired this selectivity: tangential cells now responded unselectively to a variety of moving patterns containing opposite directions of motion (Mauss et al. 2015). The tangential cells’ reduced responses to such stimuli under normal conditions can be explained by response cancelation of opponent inputs impinging on different parts of the receptive field. Motion-opponent subtraction, therefore, seems essential for flow-field selectivity in wide-field motion-sensitive neurons. This interpretation differs from previous ones where the subtraction of opponent subunits served to fix the insufficient direction selectivity of the subunit of the Hassenstein–Reichardt model (Borst and Egelhaaf 1990; Single et al. 1997). In the fly, however, this seems unnecessary, since T4/T5 cells already deliver a narrowly tuned direction-selective signal (Maisak et al. 2013).

## The core circuit underlying direction selectivity

**Fig. 5:**
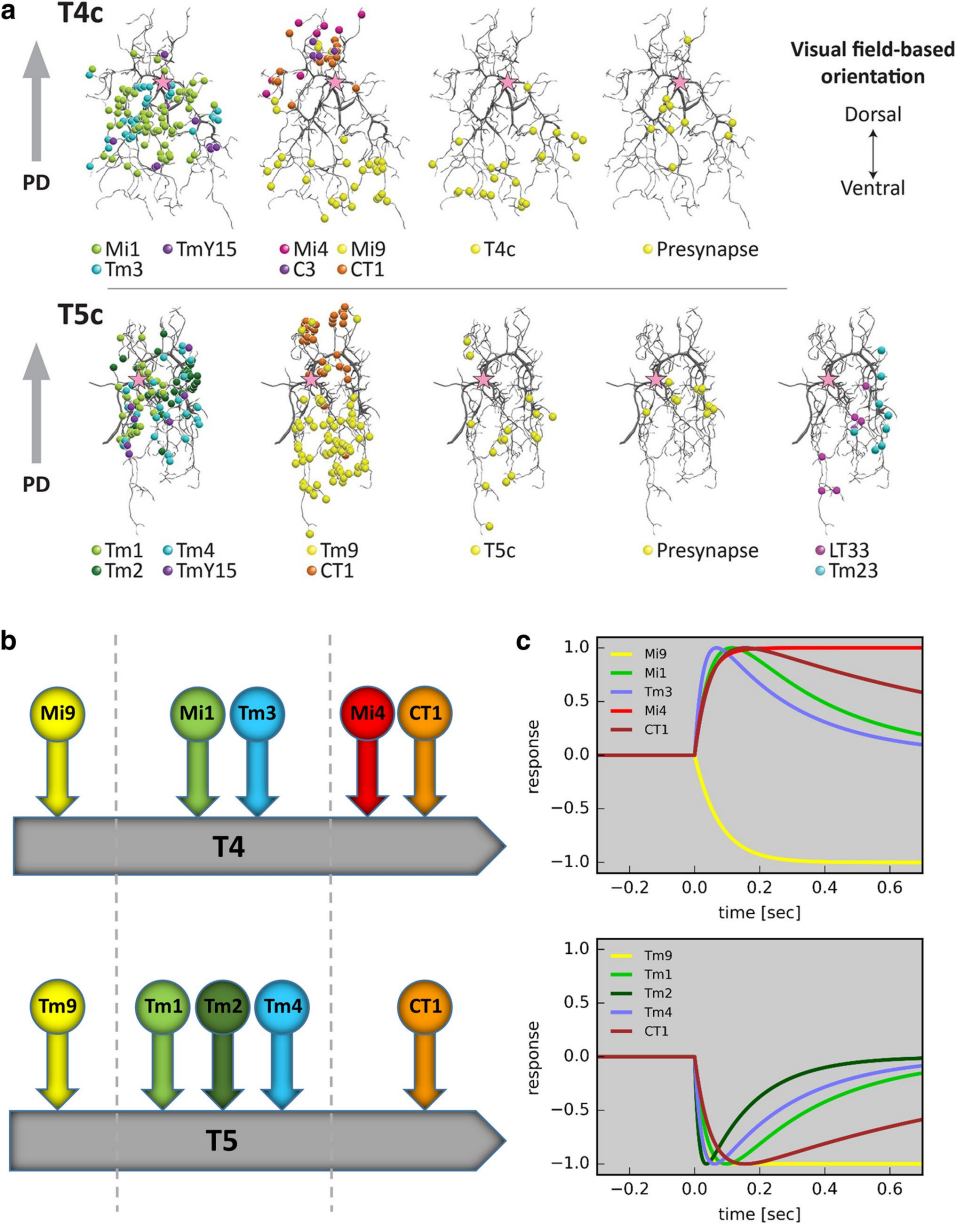
Synaptic input organization of T4 and T5 cells. **a** Spatial distribution of individual synapses of the various medulla cell types onto T4 (upper panel) and T5 (lower panel) cells, sensitive for upward motion, i.e., T4c and T5c (Shinomiya et al. 2019). **b** Schematic summary of data shown in **a**. **c** Step responses of most input cell types as derived from reverse reconstruction, using white noise stimuli and calcium imaging (Arenz et al. 2017; Meier and Borst 2019). Note that cells respond either with activity increase or decrease to stimulus onset at time point zero. Apart from the response sign, cells fall into two classes: low-pass (no response decay over time; Mi4, Mi9, and Tm9) and band-pass (after a peak, response decays back to baseline; Mi1, Tm3, CT1, Tm1, Tm2, Tm4, and CT1)

Having identified the general layout of the fly motion pathway with T4 and T5 as the elementary motion-sensing elements, the next question deals with the cells providing immediate synaptic onto T4 and T5 cells. This question was unequivocally answered by a large-scale project conducted at Janelia Research Campus (Shinomiya et al. 2019). In this project, a volume of 153 μm × 85 μm × 180 μm of the fly optic lobe comprising seven columns of the medulla, lobula, and lobula plate together with the inner chiasm connecting these neuropiles was recorded by an electron microscope (FIB-SEM, Focused Ion Beam Serial Electron Microscope) at an isotropic resolution of 8 by 8 by 8 nm. Combining machine learning algorithms for automated segmentation and synapse detection with visual inspection and proof-reading, the authors identified all the different neuron types providing inputs to the T4 and T5 cells (a subset is shown in Fig. 2). In addition, they could also locate where the different cells synapse onto the dendrite and how this differs depending on the subtype and, thus, the preferred direction, of T4 and T5 cells (Fig. 5a). The results reveal that, independent of the preferred direction, all subtypes receive input from the same medulla neurons and with the same number of synapses. For T4 cells, the presynaptic cells are Mi1, Tm3, TmY15, Mi4, Mi9, C3, and CT1. For T5 cells, the presynaptic cells are Tm1, Tm2, Tm4, TmY15, Tm9, CT1, LT33, and Tm23. In addition, each subtype of a T4 cell receives synaptic input from T4 cells of the same subtype. The same is true for T5 cells.

The dendrites of each T4 and T5 cells span several columns along the preferred direction of motion (Fig. 3d). The different subtypes differ amongst each other with respect to the column within which they receive input from a specific medulla neuron. This is exemplified in Fig. 5a for T4 and T5 cells of subtype c, i.e., the subtype that has upward motion as its preferred direction and, thus, sends its axon terminals to lobula plate layer 3. It turns out that T4c receives input from Mi1, Tm3, and TmY15 in the central part of its dendrite, from Mi9 and T4c on the ventral part and from Mi4, C3, and CT1 on the dorsal part of its dendrite (Fig. 5a, upper row). A downward selective T4d cell receives input from Mi1, Tm3, and TmY15 again in the central part of its dendrite, but from Mi9 and T4d on the dorsal and from Mi4, C3, and CT1 on the ventral part of its dendrite. As a common theme, all T4 subtypes receive Mi9 input on their preferred (i.e., the side from which a preferred direction stimulus approaches) and input from Mi4, C3, and CT1 on the null side of their dendrite (i.e., the side from which a null direction stimulus approaches). Similarly, T5 cells sample their input from Tm9 on their preferred side, Tm1, Tm2, and Tm4 in the central part, and from CT1 on their null side of their dendrite (shown for an upward selective T5c cell in Fig. 5a, lower row). Since the presynaptic cells (with the exception of CT1, see below) restrict their dendrites as well as their axon terminals to one column, this arrangement means that T4 and T5 cells sample with their multi-columnar dendrite input from adjacent points in visual space via different medulla cell types along the preferred direction axis. This is summarized in Fig. 5b, showing the tripartite input organization of T4 and T5 cells with the various cell types which they receive within each column.

Many of these input neurons have been characterized physiologically by means of calcium imaging (Meier et al. 2014; Serbe et al. 2016; Strother et al. 2017; Meier and Borst 2019), optical voltage sensors (Yang et al. 2016), glutamate sensors (Salazar-Gatzimas et al. 2018; Richter et al. 2018), or by electrophysiological recordings (Behnia et al. 2014). Importantly in the present context, none of the cells turned out to be directionally selective. Therefore, T4 and T5 cells are the first neurons within the motion pathway, which display this property and, thus, represent the important processing stage where the direction of motion is computed. Combining white noise stimulation, calcium imaging, and reverse correlation allows for determining the spatial receptive field as well as the temporal dynamics of a given neuron (Chichilnisky 2001; Ringach 2004, Clark et al. 2011; Leong et al. 2016; Salazar-Gatzimas et al. 2016; Arenz et al. 2017; Meier and Borst 2019). The analysis reveals that the input neurons fall into two classes: temporal low-pass filters and temporal band-pass filters (Fig. 5c). An interesting case is represented by the amacrine cell CT1 which arborizes within each column of the medulla and the lobula thereby covering the whole visual field (Shinomiya et al. 2015; Takemura et al. 2017). Each of these terminals shows highly compartmentalized retinotopic responses, acting as an independent functional unit, with ON responses in the medulla and OFF responses in the lobula (Meier and Borst 2019). As a common theme for both T4 and T5 cells, it emerges that the neuron on the preferred side is a low-pass filter, whereas the neurons synapsing in the center all reveal band-pass characteristics, each with its own combination of time-constants. On the null side, T4 cells receive input from at least three different cells, some of which have low-pass (Mi4; Arenz et al. 2017), others have band-pass characteristic (CT1; Meier and Borst 2019). T5 cells only receive input from a single cell (CT1) on the null side. The functional relevance of this in the context of motion vision will be discussed further below.

## The core process producing direction selectivity

**Fig. 6:**
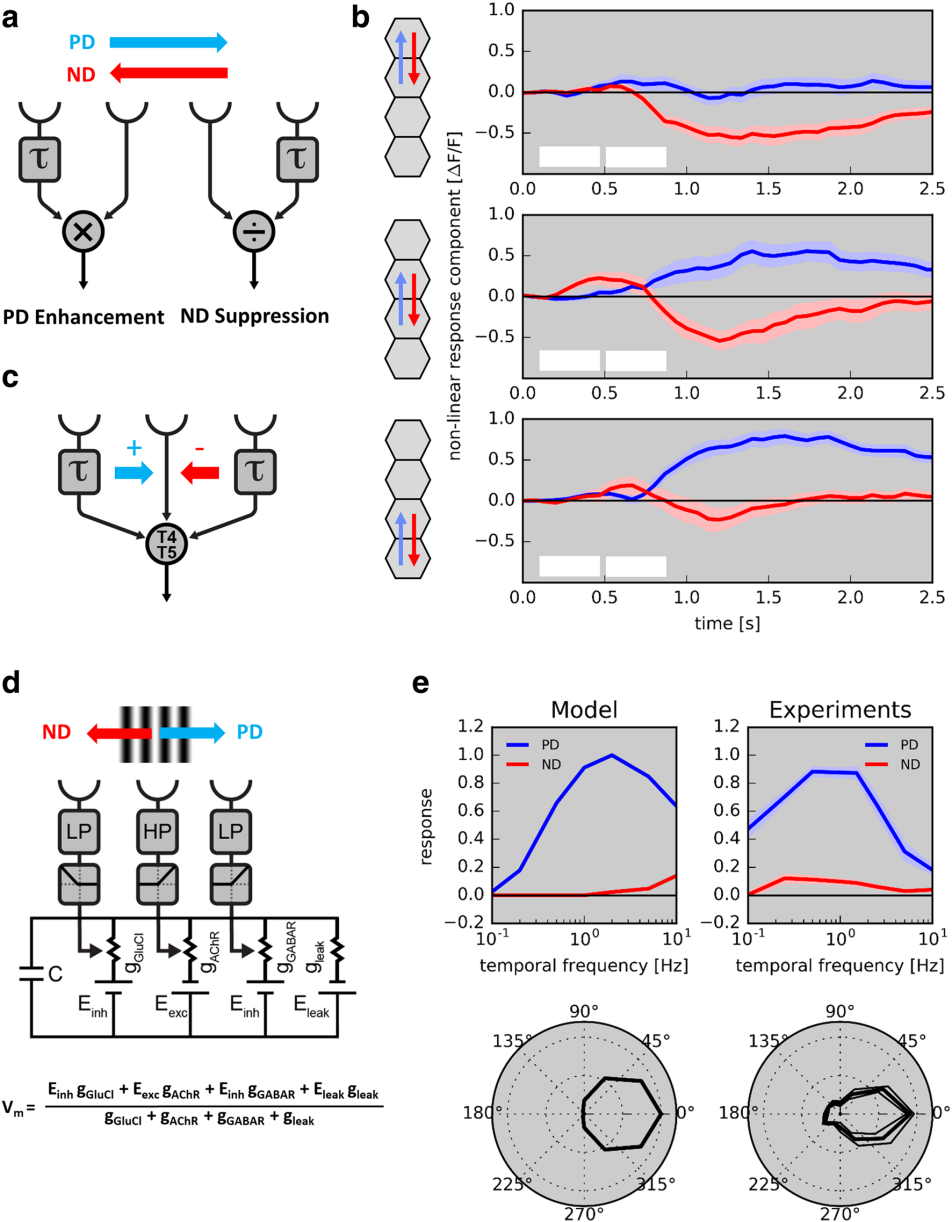
Core mechanism creating direction selectivity in T4 and T5 neurons. **a** Two alternative models to create direction selectivity: preferred direction enhancement (left) and null direction suppression (right). **b** Experiment to distinguish between the two mechanisms. Visual stimuli are placed onto the hexagonal grid of the fly eye via a telescope and responses are measured by 2P calcium imaging in upward motion-sensitive T4 cells. Two adjacent units in visual space (each unit comprising several photoreceptors ‘looking’ in the same direction) are stimulated sequentially along the preferred (blue arrows) or the null direction (red arrows) of the cell (apparent motion) or individually (no motion). First, responses to adjacent individual stimulations are shifted in time to match the time course of sequential stimulation and then summed, yielding a linear response expectation. Second, this linear expectation is subtracted from the response to the sequential apparent motion stimulation, yielding the non-linear response component. A positive deflection demonstrates a non-linear enhancement of signals (observed for the preferred direction), while a negative deflection indicates a non-linear suppression (observed for the null direction) (Haag et al. 2016, 2017). White boxes indicate the timing of sequential and individual single unit stimulation. **c** Algorithmic three-arm detector model based on calcium measurements from T4 and T5: the central input is amplified by a delayed signal from the preferred side and suppressed by a delayed signal from the null side. **d** Biophysical model implementing a three-arm model on the basis of ionic conductances (Borst 2018). Note that the left arm is an OFF element, while the central and right arms are ON elements. **e** Model performance versus experimental data (left from Borst 2018; right from Arenz et al. 2017). Top: temporal frequency tuning to gratings moving along the preferred (‘PD’, blue) and the null direction (‘ND’, red). Bottom: directional tuning to gratings moving in 12 different directions in steps of 30°

Within each subunit of the Hassenstein–Reichardt detector (Fig. 6a, left), direction selectivity is achieved by what is called ‘preferred direction enhancement’: the signal on the preferred side is delayed and, by means of multiplication, amplifies the signal from the right side. As an alternative model, the Barlow–Levick detector (Fig. 6a, right) uses a division for the non-linear processing stage. Here, the signal on the null side becomes delayed and divides the signal from the other input, leading to, what is called ‘null direction suppression’. To discriminate between these two models, apparent motion stimuli can be applied (Schuling et al. 1989; Egelhaaf and Borst 1992; Eichner et al. 2011; Fisher et al. 2015: Haag et al. 2016). Here, instead of moving an object smoothly, it is stepped discretely in space and time. For a stimulus sequence simulating preferred direction motion, the response to the second stimulus should be larger than when delivered in isolation, in case of preferred direction enhancement. For a stimulus sequence simulating null direction motion, the response to the second stimulus should be smaller than when delivered in isolation, in case of null direction suppression.

Delivering such stimuli precisely onto the optical axes of adjacent columns by means of a telescope and measuring the responses via a genetically encoded calcium indicator, Haag and colleagues found that T4 and T5 cells indeed make use of both mechanisms to produce a direction-selective response, but differently in different parts of their receptive field (Haag et al. 2016, 2017; Fig. 6b). For apparent motion sequences mimicking preferred direction motion, the first signal amplifies the second signal on the preferred side of their receptive field, with no sign of response reduction for apparent motion sequences along the null direction of the cell (Fig. 6b, lower panel). The opposite was found for stimulus sequences on the null side of their receptive field: here, the first signal reduced the second signal for apparent motion sequences mimicking null direction motion with no signs of response enhancement for apparent motion sequences along the preferred direction of the cells (Fig. 6b, upper panel). This held true for all four subtypes of T4 as well as for T5 cells (Haag et al. 2017). Consequently, a new model was proposed to account for direction selectivity in T4 and T5 cells involving both preferred direction enhancement on the preferred side and null direction suppression on the null side of the dendrite (Fig. 6c). Intriguingly, this algorithmic input structure is rather well reflected by the anatomic arrangement of presynaptic cells and their temporal filtering properties: fast Mi1 and Tm3 are positioned in the central part for the T4 dendrite, while slow neurons Mi4/CT1 and Mi9 impinge on the null and preferred flanking sides, respectively. For T5, similarly, fast Tm1, 2 and 4 are centrally located, while slow CT1 and Tm9 act on the null and preferred sides (Fig. 5b). Interestingly, using simple multiplication and division and taking the different input neurons with their distinct dynamic properties, the three-arm detector model can account for the basic response properties of T4 and T5 cells rather well. In particular and in contrast to either one algorithm in isolation, the model captures the high degree of direction selectivity that is observed in T4 and T5 cells right at the first stage where direction selectivity emerges (Arenz et al. 2017; Badwan et al. 2019).

The above-described mechanism pertains to the transformation of visual stimuli to T4/T5 calcium signals as a proxy for synaptic output. However, how are these algorithmic processes implemented biophysically, in terms of ionic conductances and membrane potential? Here, knowledge about the transmitter phenotype of the different input neurons is of importance (Takemura et al. 2017; Pankova and Borst 2017). In case of T4 cell input neurons, Mi9 on the preferred side uses glutamate (Richter et al. 2018), the two central inputs Mi1 and Tm3 use acetylcholine, and all inputs on the null side are GABAergic. Furthermore, glutamate can act as an inhibitory transmitter in the insect nervous system via a glutamate-gated chloride conductance (Liu and Wilson 2013; Mauss et al. 2015). Using these ingredients, a biophysical model to capture direction-selective changes in membrane potential was recently proposed for T4 cells (Fig. 6d; Borst 2018). It builds on the fact that the neuron providing input to T4 cell dendrite on the preferred side, Mi9, has a low-pass characteristic and an OFF center receptive field (Arenz et al. 2017). Thus, a bright edge moving along the preferred direction of T4 will first reduce the activity of Mi9 and, thus, close a chloride conductance, leading to an increase in input resistance of the T4 cell. This will lead to a larger response to the subsequent excitatory input from Mi1 and Tm3, which both have fast band-pass characteristics. For motion along the null direction, the low-pass signal from Mi4 will inhibit the subsequent excitatory central input from Mi1 and Tm3. By this way, purely based on passive membrane properties, the model can reproduce the temporal frequency profile and directional tuning of T4 cells in a rather quantitative way (Fig. 6e). However, several experimental observations seem inconsistent with this model: while dark bars elicit an increased glutamate signal on T4 cells’ dendrites, bright bars do not evoke a response decrease (Salazar-Gatzimas et al. 2018); optomotor turning responses to ON edge motion are not decreased but even slightly increased in flies with silenced Mi9 cells and GluClα knock-down in T4/T5 cells (Strother et al. 2017). Furthermore, preferred direction enhancement is not detectable in the recorded membrane potential of T4 cells (Gruntman et al. 2018).

## Open questions, controversies, and outlook

The past decade has seen enormous progress in our understanding of the motion vision circuitry in the fruit fly *Drosophila*. Given that 10 years ago, ON and OFF channels were unknown in flies and T4/T5 cell function was only subject of speculation, the present picture is full of details about the cells involved, their connectivity, and their response properties. This is indeed a satisfying situation. Nonetheless, we are still missing answers to a number of important questions.

The first question concerns the biophysical mechanism that generates preferred direction enhancement in T4 and T5 cells. In principle, a supralinearity can be produced by an increased input resistance (Koch and Poggio 1992; Borst 2018), a voltage-dependent plateau potential or similar, NMDA-like mechanisms, or a linear summation of postsynaptic potentials followed by the non-linear voltage dependence of a calcium channel (for an overview of possible mechanisms, see: Koch and Poggio 1992). If this calcium current is of negligible amplitude, it would only be visible in the calcium signal but not in the membrane voltage. As an indication for the latter, using an apparent motion stimulus protocol similar to Haag et al. (2016, 2017), Gruntman and colleagues (2018) found no indication of preferred direction enhancement in their electrophysiological recordings from T4 cells. Moreover, comparing signals from calcium and voltage indicators, Wienecke and colleagues (2018) observed neither preferred direction enhancement, nor null direction suppression in the voltage signals. The authors argued that a non-linear adaptive voltage-to-calcium transformation could account for the previously published non-linearities. Whether this is indeed the case or whether preferred direction enhancement also occurs at the level of membrane voltage but critically depends on stimulus conditions needs to be explored by future experiments. Furthermore, how is preferred direction enhancement implemented in T5 cells, with a cholinergic input on the preferred side? A final answer to these question will only come from characterizing the various currents in T4 and T5 cells, and interfering with them by either pharmacological or genetic means.

Another question of equal importance comes from the observation that both T4 and T5 cells receive input from at least 7–8 cells, including their neighboring siblings with identical directional tuning (Fig. 5a). This is in striking contrast to the two-input layout of the Hassenstein–Reichardt detector. As already mentioned, one advantage of a three-input (Fig. 6c) over a two-arm detector (Fig. 6a) is the degree of direction selectivity achieved: while in the Hassenstein–Reichardt detector, null direction responses only become annihilated after the subtraction of opponent signals, a three-arm detector is immune against null direction responses right at the first stage. But why then have T4 and T5 cells so many presynaptic inputs, and not just three? What is the specific role of each of them? At first sight, blocking experiments seem to be the way to go to provide an answer here. Indeed, in case of Mi1 and Tm3, this strategy provided first answers: blocking Mi1 abolished responses to ON edges almost completely and over a wide range of velocities, blocking Tm3 affected the responses mainly in the high velocity range. Therefore, Tm3 seems to be specifically needed to detect the direction of ON edges at high velocities (Ammer et al. 2015). However, blocking Mi9 as well as Mi4 was reported to have no effect at all on the directional behavior of flies (Strother et al. 2017). Within the OFF pathway, systematically blocking individual Tm cells as well as combinations of them revealed a range of effects on the grating and edge response, with, however, no clear indication of a specific stimulus space where the contribution of different cells would segregate (Serbe et al. 2016). There are, quite in general, three problems with the interpretation of blocking experiments: (1) Is the block effective? Without further experiments on the effectiveness of the block, negative results cannot be interpreted: either the ‘blocked cell’ has no influence on the response tested, or the block was ineffective. (2) Does the stimulus test the distinct contribution of the cell to the response? This is largely left to the intuition of the experimenter: if no phenotype is observed (and given the block is effective), maybe another stimulus would reveal the specific role of the cell under investigation. (3) Often, the outcome of blocking experiments is interpreted as if each of the input cells provides synaptic input to T4 and T5 cells in strict isolation and parallel to all the other input neurons. This, however, is not the case. Quite in contrast, almost all the different medulla neurons interact with each other extensively (Takemura et al. 2017), making the prediction for a blocking experiment far from trivial.

However, all these caveats do not represent insurmountable obstacles on the way to a full understanding of the neural circuit underlying motion vision. Therefore, with all genetic tools at hand, with all the knowledge about the connectivity, with all the genes expressed in each cell type suggesting transmitter receptors and voltage-gated ion channels, it should not take too long before the mechanism of direction selectivity is elucidated at the biophysical level, and, thus, an important and most basic neural computation is understood in unprecedented detail.
